# Headspace-Solid Phase Microextraction Approach for Dimethylsulfoniopropionate Quantification in *Solanum lycopersicum* Plants Subjected to Water Stress

**DOI:** 10.3389/fpls.2016.01257

**Published:** 2016-08-23

**Authors:** Stefano Catola, Srikanta Dani Kaidala Ganesha, Luca Calamai, Francesco Loreto, Annamaria Ranieri, Mauro Centritto

**Affiliations:** ^1^Trees and Timber Institute, National Research Council of ItalySesto Fiorentino, Italy; ^2^Department of Agriculture, Food and Environment, University of PisaPisa, Italy; ^3^Institute of Ecosystem Study, National Research Council of ItalySesto Fiorentino, Italy; ^4^School of Biology, Indian Institute of Science Education and ResearchThiruvananthapuram, India; ^5^Department of Agriculture, Food and Environmental Science, University of FlorenceFlorence, Italy; ^6^Department of Biology, Agriculture and Food Sciences, National Research Council of ItalyRoma, Italy

**Keywords:** analytical method, mass spectrometry, sulfur compounds, sampling, plant physiology

## Abstract

Dimethylsulfoniopropionate (DMSP) and dimethyl sulphide (DMS) are compounds found mainly in marine phytoplankton and in some halophytic plants. DMS is a globally important biogenic volatile in regulating of global sulfur cycle and planetary albedo, whereas DMSP is involved in the maintenance of plant-environment homeostasis. Plants emit minute amounts of DMS compared to marine phytoplankton and there is a need for hypersensitive analytic techniques to enable its quantification in plants. Solid Phase Micro Extraction from Head Space (HS-SPME) is a simple, rapid, solvent-free and cost-effective extraction mode, which can be easily hyphenated with GC-MS for the analysis of volatile organic compounds. Using tomato (*Solanum lycopersicum*) plants subjected to water stress as a model system, we standardized a sensitive and accurate protocol for detecting and quantifying DMSP pool sizes, and potential DMS emissions, in cryoextracted leaves. The method relies on the determination of DMS free and from DMSP pools before and after the alkaline hydrolysis via Headspace-Solid Phase Micro Extraction-Gas Chromatography-Mass Spectrometry (HS-SPME-GC-MS). We found a significant (2.5 time) increase of DMSP content in water-stressed leaves reflecting clear stress to the photosynthetic apparatus. We hypothesize that increased DMSP, and in turn DMS, in water-stressed leaves are produced by carbon sources other than direct photosynthesis, and function to protect plants either osmotically or as antioxidants. Finally, our results suggest that SPME is a powerful and suitable technique for the detection and quantification of biogenic gasses in trace amounts.

## Introduction

Biogenic DMS (dimethyl sulfide), a compound mainly emitted by marine environment, is a significant natural source of tropospheric sulfur (Stefels et al., 2007; Vallina and Simò, 2007; Oduro et al., 2012). DMS is important biogeochemically because its emission facilitates cycling of sulfur from the oceans to the continents (Stefels et al., 2007). Much of the DMS emitted from the marine ecosystem, mainly comprising microalgae and macroalgae, is converted to sulfate aerosol, a precursor for cloud condensation nuclei with subsequent implication for planetary albedo and, in turn, for climate change (Bates et al., 1987; Nguyen et al., 1992). DMS emission is recorded also from terrestrial ecosystems (Hanson et al., 1994; James et al., 1995) including the amazon rain forests (Jardine et al., 2015). However, in global terms, and in comparison to marine organisms, higher plants are not as significant sources of DMS and atmospheric sulfur.

**FIGURE 1:**
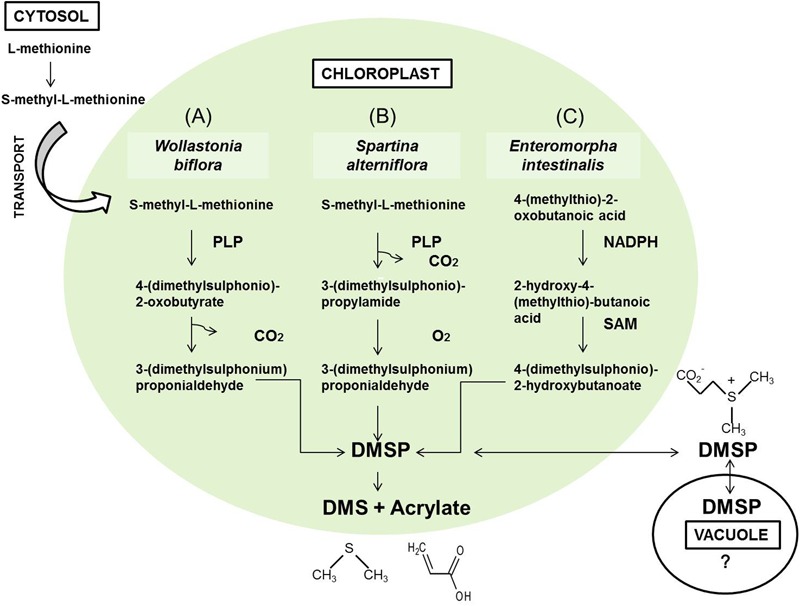
**Biosynthetic pathways of DMSP in *Wollastonia biflora***(A)**, *Spartina alterniflora***(B)** (Trossat et al., 1996), and *Enteromorpha intestinalis***(C)** (Summers et al., 1998)**.

Dimethyl sulphide is derived from the enzymatic cleavage of DMSP, an organic sulfur compound synthetized in the chloroplast from methionine imported from cytosol (Trossat et al., 1996; Gage et al., 1997; Oduro et al., 2012). Biosynthesis of DMSP proceeds three different specie-specific pathways (Kocsis et al., 1998) (**Figure 1**). Because methionine synthesis requires nitrogen and sulfur, it was shown that nitrogen availability affected the concentration of DMSP (Otte and Morris, 1994; Gage et al., 1997; Mulholland and Otte, 2002).

Various biological functions have been attributed to DMSP and DMS. Hypotheses dealing with DMSP and DMS mediated detoxification of excess sulfur, mitigation of salt and oxidative stresses, cryoprotection, and herbivore deterrence have received supportive experimental evidence (Otte et al., 2004). Abiotic stresses induce the synthesis of reactive oxygen species (ROS; Smirnoff, 1993). ROS can oxidize proteins, lipids, RNA and DNA (Cruz de Carvalho, 2008). H_2_O_2_, for example, acts as a local or systemic signal for leaf stomatal closure, leaf acclimation to high irradiance and the induction of heat shock proteins (Pastori and Foyer, 2002). DMSP, DMS, and oxidation products of DMS (dimethyl sulphoxide and methane sulphinic acid) react rapidly as scavengers of hydroxyl radical (OH). Other forms of oxidative stress including ultraviolet radiation, high radiation loads, Fe limitation, high Cu^2+^ and H_2_O_2_ lead to an increase in cellular DMSP and its lysis to DMS in marine algal cultures (Sunda et al., 2002; Rix et al., 2012). Darroch et al. (2015), for instance, showed that the synthesis of DMSP and DMS in *Emiliania huxleyi*, a coccolithophore, were upregulated under fluctuating high light and UV stresses. Under such conditions, DMS is even regarded more effective than other well-recognized antioxidants such as ascorbate and glutathione (Sunda et al., 2002).

The involvement of DMSP in osmoregulation has been accepted and demonstrated in algae and in the Pacific strand plant *Wollastonia biflora* (Storey et al., 1993; Van Bergeijk et al., 2003). DMSP could play a role as an osmotic metabolite probably due to its high chemical similarity with quaternary ammonium compounds, such as glycinebetaine, proline and known compatible organic solutes (Slama et al., 2015). It was proposed that DMSP could be involved in osmosis without changes in its concentration at tissue level in *Spartina* species (Mulholland, 2000).

Dimethyl sulphide emission in higher plants is not investigated to the same extent as marine DMS emissions and functions of DMS emission in plants are not known. The halophyte *Spartina* (cordgrass), the crop plant *Saccharum* (sugarcane), the wild mesophyte *W*. *biflora*, a moderately salt-tolerant species, and seven tropical species are reported to emit DMS (Hanson et al., 1994; Otte and Morris, 1994; Paquet et al., 1994; Jardine et al., 2015). In a rare study, *Larrea tridentata*, a mesophytic plant, was shown to emit DMS at a rate higher than any other reports of DMS emission in plants (Jardine et al., 2010). The fact that DMS emission evolved independently in different phototrophic lineages suggests different behaviors and variable functions in different organisms (Otte et al., 2004). However, the hypothesis that DMSP and its derivatives could act as antioxidant compounds and could help mitigate abiotic stresses in both phytoplankton and higher plants is the most attractive and promising (Sunda et al., 2002; Jardine et al., 2010; Darroch et al., 2015).

Sulfur volatile compounds are an important class of aroma compounds, which are often neglected because of their presence at trace levels in different higher plants. Low emission levels (close to the limits of detection of most analytical techniques) and unsuitability of sulfur containing volatiles for thermal desorption techniques (Wardencki, 1998) have hindered experiments on DMS emission in most mesophytic plants. Solid Phase Micro-Extraction (SPME) is a very fast and sensitive technique used for volatile organic compounds (VOCs) collection from foods (Pugliese et al., 2010; Calamai et al., 2012; Seisonen et al., 2015), bacteria (Tait et al., 2014), plants (Henke et al., 2015), but also for the pollutants analysis in environmental water samples (Potter and Pawliszyn, 1992). The application of headspace-solid phase micro-extraction (HS-SPME) to flavor volatiles analysis allows a very fast and simply extraction procedure that overcome the typical problems of the majority of extraction process (concentration steps, problems of memory effect and loss of most analytes). Furthermore, the flexibility of this sample approach has been employed for the determination of signal or inhibitory VOCs between microbial communities (Papaleo et al., 2012).

Preliminary analyses of potential emission of volatile hydrocarbons from tomato (*Solanum lycopersicum*), using HS-SPME-GC-MS, showed that tomato leaves emitted DMS upon alkaline hydrolysis of its leaf extracts (data not shown). Tomato is an important model species for VOCs analysis (Degenhardt et al., 2010; Ángeles López et al., 2012; Copolovici et al., 2012) and aroma of fresh tomato fruits was found to contain typical sulfur volatiles including DMS (Du et al., 2015). Our objective was to standardize a sensitive methodological protocol based on HS-SPME-GC-MS for DMSP quantification in leaf tissues of tomato plants subjected to water stress. Despite the fact that DMSP and DMS were previously determined by HS-SPME-GC-MS in seawater (Niki et al., 2004; Yassaa et al., 2006), there were no evidences in literature about DMSP determination in higher plants after its alkaline hydrolysis. Our findings are discussed in the context of potential functions of DMS emission in higher plants.

## Materials and Methods

### Plant Material

Seeds of a dwarf variety of *S. lycopersicum* L. (cv. San Marzano) were germinated in 0.7 dm^3^ pots filled with a mixture of peat:sand (1:1). Plants were grown in a growth chamber under controlled conditions (T 25°C, Photoperiod: 16∖8, RH 50%), regularly watered to full pot capacity and fertilized once a week in order to supply mineral nutrients at free-access rates until the day of the imposition of water stress (Magnani et al., 1996; Sun et al., 2008; Pallozzi et al., 2013). The experiment was performed on three week-old plants. Before the onset of the water-stress cycle, pots were wrapped with plastic bags tightly closed at the top to avoid soil transpiration and were subsequently weighed (Initial_potweight_). Then, part of the plants (*n* = 4) was water-stressed by withholding water, whereas the other part (*n* = 4) was daily watered to pot capacity. As indicator of soil water availability, the FTSW (Sinclair and Ludlow, 1986; Brilli et al., 2013) was calculated on each day as: FTSW = (Daily_potweight_ – Final_potweight_)/(Initial_potweight_ – Final_potweight_), where Daily_potweight_ is the weight of the water-stressed plants recorded during the water stress cycle and Final_potweight_ is the “pot weight” at which stomatal conductance approached zero. DMSP analysis was performed at FTSW of 30% (FTSW_30_), when stomatal conductance (*g*_s_) decreased on average about 50% of the value FTSW_100_ plants.

### Gas Exchange and Chlorophyll Fluorescence Measurements

Gas exchange and chlorophyll *a* fluorescence measurements were performed using a Li-6400 IRGA (LI-COR, Lincoln, NE, USA). A portion of a leaf was enclosed in a 2 cm^2^ cuvette, and exposed to a photosynthetic photon flux density of 700 μmol m^-2^ s^-1^, at a temperature of 25°C and with the relative humidity of the air within the cuvette ranging between 45 and 55%. By infrared gas analysis, steady-state net photosynthesis (*A*) and *g*_s_ were measured. By chlorophyll fluorescence [a sensitive indicator of PSII photochemistry (Baker, 2008)], the quantum yield of photosynthetic non-cyclic electron transport (ΦPSII *=* Δ*F*/$F_{m}^{\prime }$ = $F_{m}^{\prime }$ – *F*_s_/$F_{m}^{\prime }$) and the photochemical quenching coefficient qP = ($F_{m}^{\prime }$ – *F*_s_)/($F_{m}^{\prime }$ – $F_{o}^{\prime }$) were estimated. In the above formulas $F_{m}^{\prime }$ is the maximal fluorescence level of illuminated sample after saturating pulse, $F_{o}^{\prime }$ is the minimal fluorescence level of illuminated sample and *F*_s_ is the level of steady state chlorophyll fluorescence.

### Hydrolytic Conversion of DMSP to DMS

The same leaves, previously measured for gas exchange and chlorophyll *a* fluorescence, were photographed before alkaline hydrolysis analysis. Scanned pictures of leaves were used to calculate leaves area by using *ImageJ* software, an open source Java image processing program. DMSP contained in the specimens was hydrolyzed to DMS in accordance to the method proposed by Steinke et al. (2011), with slight modifications. An aliquot of 0.2 g for each sample was transferred to 20 ml screw cap headspace vials before adding 250 μL of NaOH (0.5 M), 2 g of NaCl and distilled water to a final volume of 5 ml. NaCl was used to favor the partitioning of volatile compounds in the headspace and improve extraction process. Headspace-vials were immediately sealed with a Teflon-coated silicone septum and incubated for 12 h at 60°C. Differently from Steinke et al. (2011) methodology, we used a different temperature for hydrolytic DMSP conversion, (e.g., 60°C for 12 h vs 30°C for 24 h) since preliminary experiments with the two conditions yielded the same results (data not reported). In addition, no further yield of DMS was obtained by protracting the hydrolysis at 60°C for additional 12 h. Based on these considerations, we performed DMSP hydrolysis in all samples at 60°C for 12 h. Immediately after incubation time, DMS was analyzed by SPME-GC-MS technique.

### Analysis of DMS Using HS-SPME-GC-MS

An Agilent 7820 GC-chromatograph equipped with a 5977A MSD with EI ionization operating at 70 eV was used for analysis. A three-phase DVB/Carboxen/PDMS 75-μm 2 cm SPME fiber (Supelco, Bellafonte, PA, USA) was exposed in the headspace of the vials at 40°C for 10 min for VOCs sampling. DMS has a boiling temperature of 37.34°C, so we used 40°C as SPME incubation temperature to favor the partitioning of DMS on the headspace for fiber’s uptake. The best extraction time was established to be 10 min, which afforded DMS uptake without causing fiber’s saturation phenomena as established in preliminary experiments. A Gerstel MPS2 XL autosampler equipped with a magnetic transportation adapter and a temperature controlled agitator (250 rpm with on/cycles of 10 s) was used for ensuring consistent SPME extraction conditions. This device ensured homogeneous sample mixing and favored the partitioning of VOCs into the headspace during SPME extraction. Chromatographic conditions were: column HP-Innowax (50 m, 0.20 mm, ID 0.4 μm DF); injection temperature 250°C, splitless mode, oven program 40° for 1 min, rate of 5°C min^-1^ to 65°C, then rate of 40°C min^-1^ to 260°C held for 5 min. Mass spectra were acquired within the 29–350 M/Z interval with an Agilent 5977 MSD spectrometer at a scan speed such to obtain three scans s^-1^. The identification of volatile DMS was done by matching the peak spectrum with library spectral database (NIST 11.L) and by comparing retention times and mass spectrum with those of an injected authentic standard, DMS standard was purchased by Sigma-Aldrich (Milano) and its assay was ≥98%.

**FIGURE 2:**
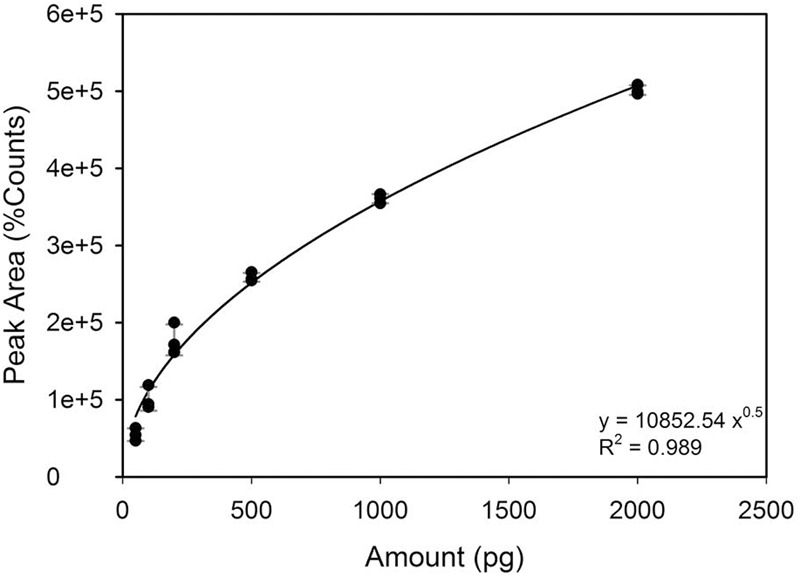
**DMS calibration curve obtained by replicating each level of calibration three times ±SD**.

**FIGURE 3:**
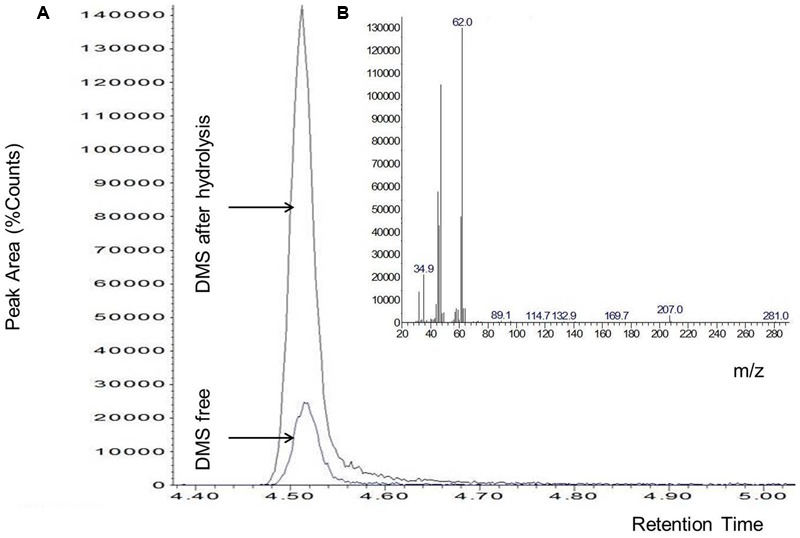
**Chromatogram of DMS without (DMS free) and with alkaline hydrolysis of DMSP **(A)**, characteristic spectrum of DMS **(B)**.** Data shown are from a single leaf but are representative of experiments replicated four times on different plants per water treatment.

**FIGURE 4:**
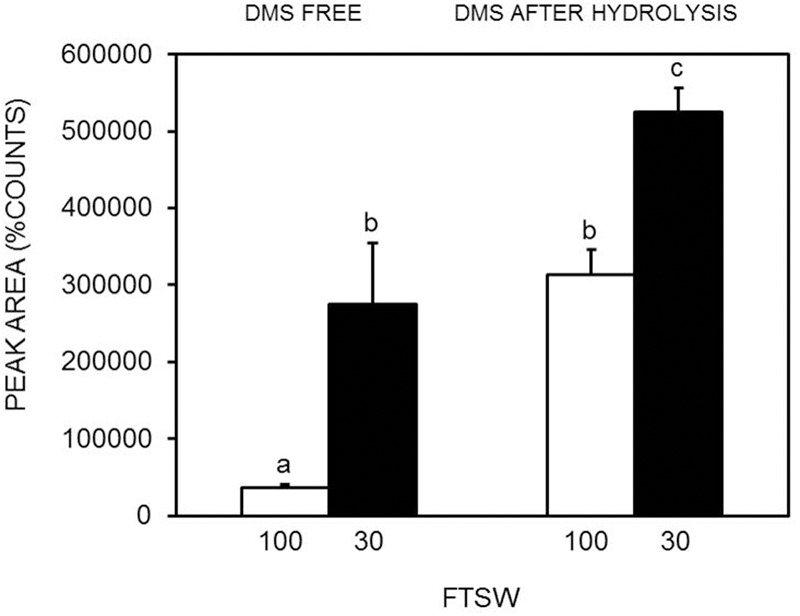
**Comparison of DMS free (without DMSP hydrolysis) and DMS after alkaline hydrolysis in well-watered (FTSW_100_) (white bar) and water-stressed (FTSW_30_) (black bar) *S. lycopersicum* leaves.** Data are means of four replicates ±SE. Letters indicate differences at *P* < 0.05

The DMS standard was also used to quantify DMSP with a calibration curve in the range of 50–2000 pg (i.e., the amounts range found in the specimens) (**Figure 2**). Control analyses on specimens before hydrolysis, indicated that the amounts of free DMS eventually present was negligible as compared to DMS after hydrolysis (**Figure 3**). On the other hand, a comparison of the “free DMS” between well-watered and water-stressed plants revealed that “free DMS” in water-stressed leaves was significantly higher compared to that of well-watered leaves, albeit the amounts were negligible as compared to the DMS recovered after alkaline hydrolysis (**Figure 4**). The limit of detection (LOD), the limit of quantification (LOQ) were calculated as reported by Shrivastava and Gupta (2011). Particularly LOD and LOQ were calculated based on standard deviation of response peak and slope. Accuracy (measured value/expected value) and precision (±standard deviation) were determined in the extreme levels of calibration range.

### Statistical Analysis

Physiological data and DMS variations were tested using student’s *t*-test. Comparison of DMS free and DMS derived by alkaline hydrolysis in well-watered and water-stressed leaves were tested using one-way ANOVA with Tukey *post hoc* test. Statistics and figures were performed by Sigma Plot 12.5 (Systat software, Inc., San Jose, CA, USA).

## Results and Discussion

### Photosynthetic Traits

**FIGURE 5:**
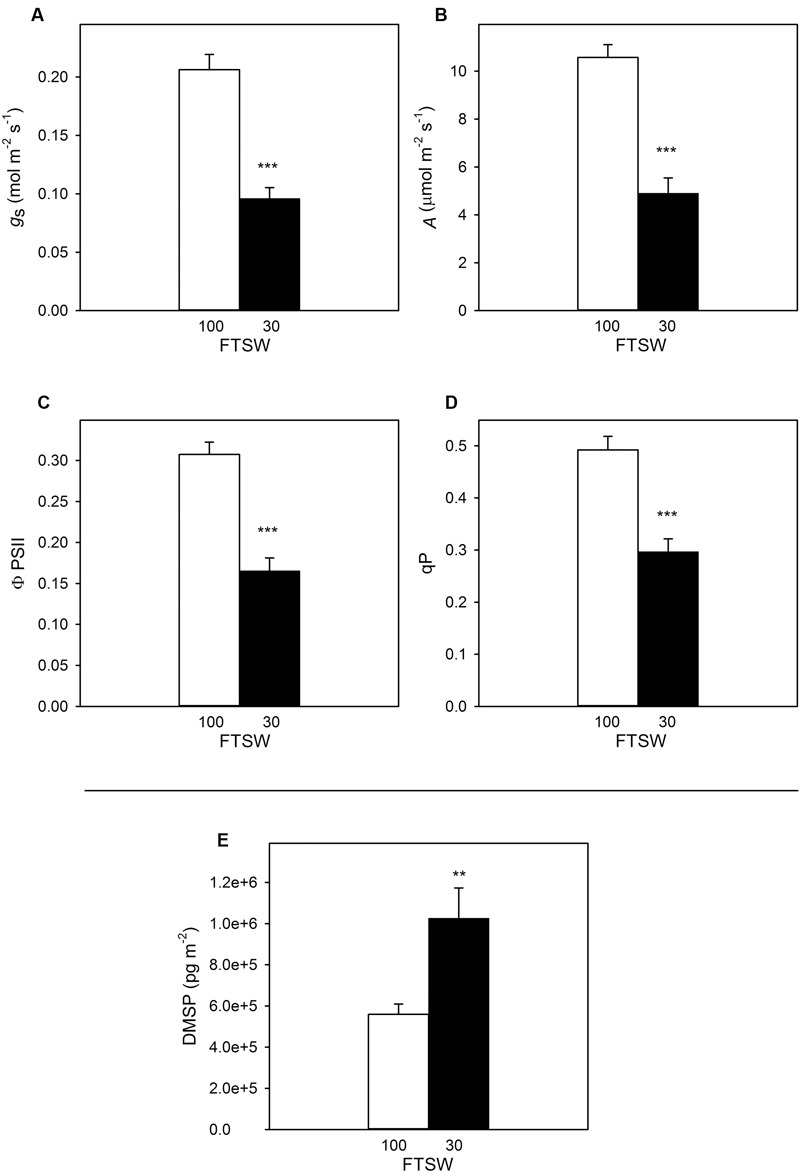
**Stomatal conductance (*g_s_*) **(A)**, photosynthesis (*A*) **(B)**, quantum yield of photosynthetic non-cyclic electron transport (ΦPSII) **(C)**, photochemical quenching (qP) **(D)** and DMSP concentration **(E)** in well-watered (FTSW_100_) (white bar) and water-stressed (FTSW_30_) (black bar) *S. lycopersicum* leaves.** Data are means of four replicates ±SE. Asterisks indicate differences at *P* < 0.01 (^∗∗^) and *P* < 0.001 (^∗∗∗^).

During the time course of the experiment *A*, ϕPSII and qP showed a slight, non-significant decline in well-watered plants (FTSW_100_), but *g*_s_ (the indicator used to assess stress level) remained stable at FTSW_100_ (data not shown). As expected (Aganchich et al., 2009; Centritto et al., 2011; Brilli et al., 2013; Marino et al., 2014), all the photosynthetic traits were significantly affected by water stress (**Figure 5**). Stomatal conductance decreased from 0.20 ± 0.20 to 0.09 ± 0.01 mmol s^-1^ (∼50% of the initial value) (**Figure 5A**), whereas *A* dropped from 11 ± 0.86 to the value of 4.9 ± 0.64 μmol m^-2^s^-1^ when the FTSW of water-stressed *S. lycopersicum* pots reached 30% compared to FTSW_100_ (**Figure 5B**). Similarly, at FTSW_30_, ϕPSII (**Figure 5C**) and qP (**Figure 5D**) had a reduction of approximately 54 and 60%, respectively, compared to FTSW_100_ plants.

### Water Stress Increases DMSP Content in the Leaves of *S. lycopersicum*

We have developed a sensitive and reliable methodology to detect DMSP and DMS in higher plants. The DMSP amount, expressed on a leaf area basis (pg m^-2^), was determined by using the calibration curve of DMS (**Figure 2**).

The DMSP content retrieved in FTSW_30_ plants significantly increased by 2.5 times compared to well-watered plants (i.e., FTSW_100_), and statistical analysis performed showed significant differences between the two treatments with a *P* < 0.01 (**Figure 5E**).

In general, DMS emission by plants has been elusive, and only occasionally reported (Hanson et al., 1994; James et al., 1995; Jardine et al., 2015). Emissions of DMS have been generally attributed to marine photosynthetic organisms (Sunda et al., 2002; Darroch et al., 2015), but our report shows that also higher plants can produce DMSP and potentially emit DMS. However, we confirm that DMSP content in plants, such as in our tomato samples is in the order of pmol, i.e., 5–6 times less than DMSP determined in marine algae (Sunda et al., 2002). By using this novel methodology, we were able to detect a strong and positive effect of water stress on DMSP biosynthesis.

Although we did not directly measure DMS, the reliable and steady conversion of DMSP into DMS is an indication that water-stressed plants may release larger shares of the latter volatile. Despite the fate and role of terrestrial DMS emission in the atmosphere remains to be studied, it is likely that this emission may also have consequences on atmospheric pollution, as it happens for DMS emitted in marine environment (Bates et al., 1987; Nguyen et al., 1992). In algae, DMS emission increases with increasing temperature as more DMSP gets converted into DMS (Arnold et al., 2013). Thus, one would expect that DMS emission by terrestrial plants may largely increase as a function of rising air temperature, perhaps even more than in marine environments where temperature increases are buffered by the water. As stomata close due to water stress, less latent heat is released by evapotranspiration resulting in increased leaf temperature (Siddique et al., 2000). Consequently, also water stress should cause larger conversion of DMSP into DMS, and consequently larger emissions of DMS. However, our experiment shows that the entire biosynthesis of DMSP is stimulated under water stress. Metabolically, this may represent enhanced contribution of carbon sources other than carbon freshly fixed by photosynthesis, as photosynthetic activity was largely inhibited by water stress. A labeling experiment would help clarifying whether additional carbon sources are used for DMSP biosynthesis in stressed leaves, as is the case for isoprene (Brilli et al., 2007). The concentration of DMSP was reported to increase with increasing salinity in *Spartina alterniflora* (Mulholland and Otte, 2002). The initial responses to water and salt stress are considered to be similar (Munns, 2002), because both stresses induce osmotic adjustment for the maintenance of water uptake and cell turgor under water deficit. The accumulation of DMSP in water-stressed leaves could, thus, indicate an osmoprotective function of this compound as previously suggested (Mulholland and Otte, 2002; Slama et al., 2015). However, it remains to be tested whether DMSP moves between cytoplasm and vacuoles within the cells (Mulholland, 2000), and whether a similar mechanism can help maintain water potential during water stress.

Increase in DMSP content and – supposedly – DMS emission might be also associated with their antioxidant function. An antioxidant role was proposed for these molecules in marine algae (Sunda et al., 2002) and then also in higher plants (Jardine et al., 2010). In the case of DMSP, this action could be exerted especially by its oxidation products (e.g., dimethylsulphoxide, DMSO) which are further able to react with and to scavenge OH radicals (Sunda et al., 2002). The chemical structure and high volatility of DMS makes this volatile molecule also a potential antioxidant in most biological systems. DMS may also be oxidized to DMSO (Sunda et al., 2002). The functions of DMS may also be reminiscent of those of another volatile molecule (isoprene) in higher plants. DMS, alike isoprene (Loreto and Velikova, 2001; Velikova et al., 2012), may readily diffuse into and beyond cellular membrane, therefore acting as a delocalized antioxidant, and intervening at locations where ROS removal is most needed. Based on findings presented here, i.e., stimulation of DMSP synthesis and absence of isoprenoid emission in water-stressed tomato plants, we speculate that isoprene and DMS constitute to evolutionary alternative but functionally similar way of coping with stress. Both marine organisms and higher plants might have evolved one of the two protective mechanisms, but rarely both together, due to the expensive investment in carbon and energy in these molecules (Sharkey and Yeh, 2001).

### Headspace-Solid Phase Micro Extraction-Gas Chromatography-Mass Spectrometry Method for DMSP Quantification

The LOD, the LOQ, the accuracy and the precision of the calibration curve were reported in **Table 1**. The calculated LOD and LOQ values were as low as 32.2 and 107.6 pg, respectively, i.e., lower than the amounts actually measured in either stressed and not-stressed specimens. In fact, the amounts of DMSP in tomato leaf extracts of well-watered and water-stressed plants were 556.26 (±142.29) and 1025.29 (±96.27) pg, respectively. Consequently, the calibration curve obtained by SPME technique allowed estimating DMSP content in *S. lycopersicum* leaves with an accuracy, calculated in the highest levels of calibration range, of 97.7% (**Table 1**). SPME DVB/Carboxen/PDMS fibers efficiently captured DMS from the headspace vials at the selected experimental conditions allowing good reproducibility of results. A possible limitation of this technical approach is its application for quantitative analysis, because some phenomena (wearing of fiber’s coating, fiber saturation at the highest concentrations, and competition of multiple analytes) may occur along with the experiments and, as a consequence, alter the linearity of calibration response. On the other hand, fiber saturation phenomena can be eventually corrected by using suitable internal standards (Calamai et al., 2012). As an alternative, not linear calibration functions may be used, such as in the present paper (**Figure 2**). This may of course represent a problem, if the amounts of DMS in samples are close to the LOD (32.2 pg), which is not our case. As shown in **Table 1**, at the lowest calibration levels, the accuracy of the methodology was not as good (49.7%) as at highest calibration levels (97.7%), despite an high precision along the of calibration range.

**Table 1:** Accuracy and precision of the HS-SPME method determined at the extreme range of the calibration curve, showed in **Figure 2**.

LOD (pg)	32.2
LOQ (pg)	107.6
Accuracy (%) Low calibration level	49.7
Accuracy (%) High calibration level	97.7
Precision (±SD) Low calibration level	±7.3
Precision (±SD) High calibration level	±47.8

*LOD, limit of detection; LOQ, limit of quantification, calculations were based on the SD of response peak and curve slope (Shrivastava and Gupta, 2011).*

## Conclusion

The Headspace-Solid Phase Microextraction (HS-SPME) technique used for assessing both DMSP content and the potential emission of DMS in tomato resulted highly effective in the detection and quantification of these compounds. DMSP and DMS are mostly found in marine ecosystems and in halophytic plants, where they serve a protective function against different abiotic stresses. In our knowledge, this study is the first to report DMSP and DMS in higher plants such as tomato. Using this novel, powerful methodology, we found a strong positive effect of water stress on DMSP content in tomato leaves experiencing an inhibition of their photosynthetic apparatus. These results suggest a potential functional role for DMSP and for its volatile product DMS in terrestrial vascular plants.

## Author Contributions

SC, LC, and MC designed the research. SC and LC performed the experiment; SC, LC, and SK analyzed data; All authors contributed to write the manuscript.

## Conflict of Interest Statement

The authors declare that the research was conducted in the absence of any commercial or financial relationships that could be construed as a potential conflict of interest.

## Abbreviations

DMS: Dimethyl sulphide
DMSP: Dimethylsulfoniopropionate
FTSW: Fraction of Transpirable Soil Water
HS-SPME-GC-MS: Headspace-Solid Phase Micro Extraction-Gas Chromatography-Mass Spectrometry
VOCs: Volatile Organic Compounds
